# Concomitant screening of coronary artery disease and lung cancer with a new ultrafast-low-dose Computed Tomography protocol: A pilot randomised trial

**DOI:** 10.1038/s41598-019-50407-6

**Published:** 2019-09-25

**Authors:** Carlo Gaudio, Alessandra Tanzilli, Mariachiara Mei, Andrea Moretti, Francesco Barillà, Antonio Varveri, Vincenzo Paravati, Gaetano Tanzilli, Antonio Ciccaglioni, Stefano Strano, Massimo Pellegrini, Paolo Barillari, Francesco Pelliccia

**Affiliations:** 1 Department of Cardiovascular Sciences, Sapienza University, Rome, Italy; 2 Villa Mafalda Clinical Institute, Rome, Italy

**Keywords:** Cardiology, Cancer

## Abstract

We performed a pilot randomised study to assess the feasibility and radiation exposure of a new computed tomography (CT) protocol that allows screening of both coronary artery disease (CAD) and lung cancer. Current or former heavy smokers at high lung cancer risk with indication to cardiac CT for suspected or known CAD were randomised to undergo concomitant CT evaluation of either cardiac or thoracic area or cardiac CT only. Out of 129 subjects deemed eligible for the study, 110 agreed to participate and were randomised to simultaneous cardiac and lung CT (Gr.A; n = 55) or cardiac CT only (Gr.B; n = 55). The feasibility (i.e. adequate visualization of coronary artery segments) was noninferior with simultaneous cardiac and lung CT compared with the standard cardiac CT (870 of 889 segments [97%] in Gr.A vs 878/890 segments [99%] in Gr.B; mean difference 2.0% [90% confidence interval: −0.3% to 4.1%]). The safety (i.e. effective radiation dose) of the concomitant cardiac and lung CT protocol was noninferior to the standard cardiac CT (1.5 [95% confidence intervals: 1.2–1.7] vs. 1.4 [95% confidence intervals: 1.1–1.6] mSv; mean difference 0.1 mSv [90% confidence interval: −0.2 to 0.3 mSv]). In the two groups, a total of 25 significant (>70%) coronary stenoses were found at cardiac CT (9/55 cases of Gr.A vs 11/55 cases of Gr.B). Pulmonary nodules >2 mm were detected in 7 of the 55 Gr.A subjects. This pilot randomised study shows that concomitant CAD and lung cancer screening by means of a new CT protocol is both feasible and safe, thus allowing a comprehensive evaluation of both cardiac and thoracic regions during one CT scanning only. (ClinicalTrials.gov Identifier: NCT03727958).

## Introduction

Computed tomography (CT) has rapidly become an important technique in the diagnostic work-up of coronary artery disease (CAD). The update edition of the National Institute for Health and Care Excellence (NICE) guidelines state that cardiac CT is the non-invasive test of choice in the evaluation of patients with stable angina in order to rule out CAD^1^. Also, patients with known CAD who previously had percutaneous coronary intervention might benefit from cardiac CT in case of recurrent angina or diagnostic findings indicating that a ‘de novo’ stenosis might have occurred^2^. Noteworthy, patients with suspected or known CAD often have a high lung cancer risk as well^3^. Screening of lung cancer with CT is recommended since 2014 in subjects who are current or former heavy smokers whose age range between 55 and 80 years^4^. Accordingly, high risk subjects with indication to cardiac CT need also lung cancer evaluation in order to accomplish current recommendations on screening of lung cancer^5^.

We recently proposed a CT protocol for the simultaneous screening of CAD and lung cancer in those subjects who are deemed to be at high risk for the two conditions^6^. Our preliminary data obtained from 30 subjects showed that the new protocol reliably allows concomitant cardiac and lung CT scanning, thus avoiding to double the doses of radiation and contrast dye which are used when two distinct examinations are performed to study the coronary arteries and the pulmonary areas. It remains unknown, however, if the new protocol might affect the evaluation of coronary artery segments and radiation exposure. To address these important points, we performed a pilot randomised study aimed at assessing the feasibility and safety of the new approach as compared with the standard cardiac CT.

## Methods

This investigation was disegned as an open-label, randomised, noninferiority pilot study. Study partecipants were recruited from the Outpatient Clinic of the Department of Cardiovascular Sciences of the Sapienza University, Rome, Italy, where approximately 10,000 ambulatory subjects are referred every year. All underwent CT examination in an hospital-affiliated imaging center. A consecutive series of current or former heavy smokers whose age ranged between 55 and 79 years were screened for eligibility. The study was conducted according to the principles of the Declaration of Helsinki, and was performed in accordance with current scientific guidelines. Also, the protocol was approved by the Institutional Review Board Committee of the Department of Cardiovascular Sciences of the Sapienza University, Rome, Italy, (No. 2018/D/456). Written informed consent was obtained from each study partecipant. The STARD (Standards for Reporting of Diagnostic Accuracy Studies) guidelines for publishing investigations on the diagnostic accuracy were adopted^7^. Also, informed consent for data sharing and image publication was obtained from all partecipants. The study was registered with the National Clinical Trials Registry (ClinicalTrials.gov Identifier: NCT03727958).

### Study population

The inclusion criteria were: (1) current or former habit of heavy smoking (i.e. at least 30 pack-years of smoking); (2) age ranging from 55 to 79 years; (3) both spontaneous and exercise*-*induced chest pain; (4) written informed consent to undergo CT scanning. Exclusion criteria were: (1) contraindications to contrast agent, including chronic renal failure - as defined by an estimated glomerular filtration rate lower than 60 ml/min/1.73 m^2^ - or history of allergic reactions; (2) microalbuminuria; (3) diagnosis of acute coronary syndrome; (4) irregular heart rate; (5) any suspicion of pregnancy; (6) inability to provide informed consent.

All eligible subjects willing to participate to the study were randomised in a 1:1 fashion to have cardiac CT only for ruling out CAD or to undergo simultaneous cardiac and lung CT assessments for screening CAD and lung cancer. Invasive coronary angiography (ICA) was performed afterwards when patients had evidence of at least 1 significant (i.e. reduction > 70% in vessel diameter) stenosis in a coronary artery.

### CT examinations

All examinations were performed with a Revolution CT system (General Electric, Boston, MA, US) (Table 1). When heart rate was higher than 70 beats/min, the administration of beta-blockers (at least 50 mg of metoprolol) was recommended in order to obtain heart rates <60 beats/min prior to CT scanning. In subjects randomised to undergo cardiac CT only, standard prospectively ECG-triggered sequential cardiac images were obtained. In those randomised to have combined cardiac and lung CT evaluation, a new protocol was used^5^. A prospectively ECG-triggered SnapShot Pulse™ acquisition - which allows a significant radiation dose reduction (up to 82%) using the ASiR-V™ iterative reconstruction algorithm - started from the carena to the apex of the heart to evaluate coronary arteries (100 kVp, variable mAs, thickness 0,625 mm, field-of-view 16 mm), and an additional second fast, low dose scan of the whole chest, from pulmonary apex to the bases (100 to 120 kVp, auto mAs to adapt to the patient body mass index, thickness 1,25 mm, field-of-view 24–28 cm) were performed. A single bolus (60–100 mL) of iodinated contrast agent (Ultravist™, Bayer HealthCare Pharmaceuticals, Berlin, Germany) was used in all examinations.

**Table 1:** Technical characteristics of CT scanning protocols.

Parameters	Cardiac and lung CT protocol	Cardiac CT protocol
**Cardiac CT**		
Scan type	Axial	Axial
Cardiac acquisition	Prospective	Prospective
Collimation (mm)	40 mm	40 mm
Slice thickness	0.625 mm	0.625 mm
SFOV	Cardiac large	Cardiac large
DFOV	16 mm	16 mm
Rotation speed	0.28 s	0.28 s
Tube current	Automatic (depending on BMI)	60 mA
Tube voltage	100 KVp	120 KVp
Current modulation	Step-and-shoot acquisition *(“SnapShot Pulse” algorithm*)	ECG driven mA modulation
**Lung CT**		
Tube current	Automatic (depending on BMI)	—
Tube voltage	100 to 120 KVp	—
Slice thickness	1.25 mm	—
SFOV	Chest large	—
DFOV	24–28 cm	—

BMI = body mass index; CT = computed tomography; DFOV = Display field of view; SFOV = Scan field of view.

### Radiation dose estimates

Contrast-to-noise ratio (CNR) and signal-to-noise ratio (SNR) were derived for each examination. Protocols for CT acquisition allowed to estimate radiation doses during every CT scan. In every patient, the dose-length product (DLP) was assessed. Also, the effective radiation dose (ED) was measured in each case with the formula “ED (mSv) ≈DLP × k”, where k is a conversion coefficient specific for adult chests (0.014 mSv/mGy × cm)^8^.

### CT images analysis

Lung image interpretation was performed by two radiologists (MP and PB) specifically trained in thoracic imaging. Pulmonary nodules were analysed according to the National Comprehensive Cancer Network (NCCN) guidelines for lung cancer screening^9^. Nodules were defined as rounded or irregular opacities, well or poorly defined, measuring up to 2 cm in diameter. Those with homogenous soft-tissue attenuation were characterised as solid nodules, and those presenting hazy increased attenuation, within which margins of pulmonary vessels could be indistinct, as ground-glass nodules. Positive results required the identification of a non calcified solid nodule ≥6 mm or a ground-glass nodule >5 mm^9^.

Cardiac image analysis was done by two blinded readers (AT and MM). Assessment of coronary stenoses was carried out in 16 coronary artery segments, plus the intermediate branch if present (i.e., segment 17), according to the classification of the American Heart Association^10^. The analysis was performed using the cardiac CT workstation’s specialised software (Vitrea2 FX, Vital Images, Plymouth, Minnesota). Briefly, the operators used the vessel detection tool available with the workstation, which allows the automatic creation of curved multiplanar reformations along the coronary arteries. Also, the maximum-intensity projections and the so-called ‘cath’ views were obtained^11^. Image quality of CT was graded independently by both readers on a 4-point ordinal Likert-type scale, where 1 nondiagnostic, 2 moderate, 3 good, and 4 excellent visibility). Both readers first determined how segments were visualised and then categorised the severity of diameter stenosis as being <70% or 70% or greater^12^. Percentage of assessable coronary segments was calculated.

### Quantitative coronary angiography

Quantitative ICA was performed by operators that were unaware of the study protocol (AV, GT, and FP) and was regarded as the gold-standard method. Left and right coronary angiography was performed in multiple views within one week of CT. Quantitative ICA allowed identification of arteries with significant flow-limiting lesion as defined by a >70% diameter stenosis. Digital angiograms were analysed off-line with the use of an automated edge-detection system (Cardiovascular Medical System, MEDIS Imaging Systems, Leiden, The Netherlands)^13^. All measurements were performed on cine-angiograms recorded after nitroglycerin administration. All visible lesions, including wall irregularities, were analysed on the angiograms. Multiple lesions within one coronary artery segment were considered distinct whenever separated by a visually smooth arterial wall. The measurement of percent diameter stenosis was performed in the projection showing the highest degree of narrowing. The contrast-filled non tapered catheter tip was used for calibration, and the reference diameter was measured by interpolation. At baseline, all segments >2 mm in diameter with a >20% diameter stenosis were measured^13^. Percent diameter stenosis was calculated as (reference diameter-minimal luminal diameter)/(reference diameter) × 100).

### Primary and secondary outcomes

The primary outcome of the study was twofold: (a) equivalence of the feasibility of the new protocol (i.e. the number of coronary artery segments adequately assessable in the two groups); (b) equivalence of the safety of the new protocol (i.e. the effective radiation doses in the two groups). The secondary end-points of the study were: (a) inter-observer variability in image quality of cardiac CT images in the two groups; (b) inter-observer variability in diagnosis a coronary artery stenosis >70% at cardiac CT scanning in the two groups; (c) agreement in detecting a coronary artery stenosis >70% between cardiac CT examination and ICA as assessed in the subset of patients who underwent both procedures.

### Statistical analysis

This study was designed to test the equivalence of the combined cardiac and lung CT protocol vs. conventional cardiac CT protocol with Δ = 15% on the basis of previous observations^6^. Sample size calculation was performed at time of study design in order to avoid any “*post-hoc*” power calculation. Calculation determined that 106 patients (i.e. 53 patients in each arm [95% Confidence Interval: 51.6 to 54.4]) were required to be 80% sure that the limits of a two-sided 90% confidence interval will exclude a difference in means of more than 15% for the new CT protocol compared with the reference cardiac CT protocol^14^. Equivalence was established when the mean differences between primary end-points lied within the prespecified non-inferiority zone^15^. The agreement between observers for image quality and diagnosis ability of CT protocols as well as the agreement between CT and ICA were based on the Pearson correlation coefficient (i.e. a value >0.80 of κ indicated an excellent level of agreement). Differences in baseline clinical and angiographic characteristics of the study patients was compared with descriptive statistics. The differences between normally distributed continuous values were assessed using an unpaired two-tailed Student’s t-test or one-way analysis of variance. The differences between categorical variables were compared by chi-square test or Fisher’s exact test, when appropriate. Statistical analysis was performed with R software 3.4.0 (The R Foundation for Statistical Computing). A p ≤ 0.05 was considered statistically significant.

## Results

**Figure 1:**
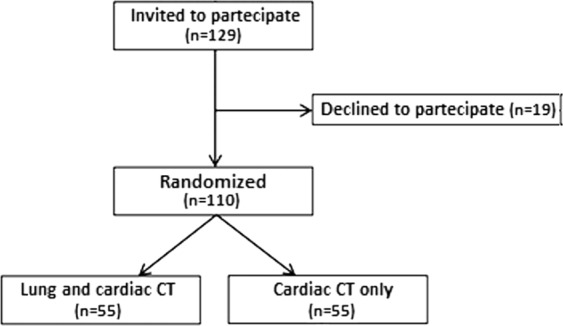
Flow-chart of the pilot trial indicating patient recruitment and allocation of study population.

A total of 129 current or former heavy smokers with age ranging between 55 and 79 years were consecutively deemed eligible and were invited to enter the study. Nineteen subjectes declined to participate (acceptability: 85%) and therefore the study sample consisted of 110 individuals (74 men and 36 women, mean age: 67 ± 10 years). The study population was randomised to lung and cardiac CT (Gr.A; N = 55 subjects) or to cardiac CT only (Gr.B; N = 55) (Fig. 1). Comparison of the baseline demographic and clinical characteristics between the two groups did not show any significant difference (Table 2).

**Table 2:** Baseline clinical and angiographic characteristics of the study patients.

	Cardiac and lung CT (Gr.A; n = 55)	Cardiac CT only (Gr. B; n = 55)	P
Age (years)	69 ± 9	65 ± 8	0.992
Male sex	39 (71%)	35 (64%)	0.416
Body mass index (kg/m^2^)	22.1 ± 3.1	23.4 ± 2.5	0.978
**Medical history**			
Current smoking	24 (45%)	25 (20%)	0.436
Hypertension	29 (53%)	30 (54%)	0.848
Total cholesterol > 200 mg/dl	38 (69%)	40 (73%)	0.674
Diabetes mellitus	12 (21%)	13 (24%)	0.820
History of angina	28 (51%)	27 (49%)	0.848
Previous diagnosis if CAD	12 (22%)	15 (27%)	0.506
ASCVD risk score (%)	10.1 ± 6.8	9.6 ± 3.8	0.864
**Laboratory data**			
LV ejection fraction (%)	56 ± 19	52 ± 16	0.882
Blood creatinine (mg/dL)	1.22 ± 0.50	1.36 ± 0.64	0.898
eGFR (mL/min/1.73 m^2^)	0.85 ± 0.17	0.93 ± 0.19	0.989
Total cholesterol (mg/dL)	194 ± 75	208 ± 69	0.844
Triglyceride (mg/dL)	169 ± 81	181 ± 79	0.783
**Concomitant medications**			
Beta-blockers	18 (32%)	20 (36%)	0.688
ACE-inhibitors/ARBs	25 (45%)	23 (41%)	0.700
Statins	29 (53%)	27 (49%)	0.703

Values are number of patients (%) or mean ± SD. ACE = angiotensin converting enzyme; ARB = Angiotensin receptors blocker; ASCVD = atherosclerotic cardiovascular disease; CAD = coronary artery disease; CT = computed tomography; eGFR = estimated glomerular filtration rate; LV = left ventricular.

### Feasibility and safety outcomes

**Figure 2:**
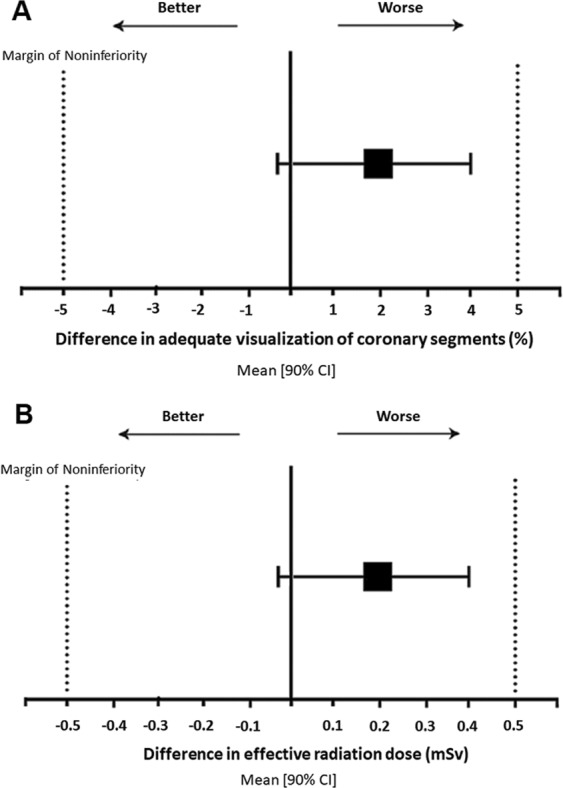
(**A**) Equivalence of concomitant cardiac and lung CT protocol in the feasibility end-point. The mean difference [90% confidence intervals] in proportion of adequate visualization of coronary artery segments between the two CT scan protocols lied within the margins of non-inferiority; (**B**) Equivalence of concomitant cardiac and lung CT protocol in the safety end-point. The mean difference [90% confidence intervals] in effective radiation dose between the two CT scan protocols lied within the margins of non-inferiority.

The primary outcome of the study was twofold, i.e. the feasibility and the safety of the new protocol. The coronary segments were assessable in 103/110 subjects, 51 (93%) of Gr.A and 52 (95%) of Gr.B. No step artefacts were observed in the two groups. Conversely, motion artefacts occurred in 7/110 (6%) cases thus compromising assessability of 31 out of 1,779 coronary segments. The feasibility end-point (i.e. adequate visualisation of coronary artery segments) showed equivalence between combined cardiac and lung CT and the standard cardiac CT (870 of 889 segments [97%] in Gr.A vs 878/890 segments [99%] in Gr.B; mean difference 2.0% [90% confidence interval: −0.3% to 4.1%]) (Fig. 2). The safety end-point (i.e. effective radiation dose) was equivalent between the cardiac and lung CT protocol and the standard cardiac CT (1.5 [95% confidence intervals: 1.2–1.7] vs. 1.4 [95% confidence intervals: 1.1–1.6] mSv; mean difference 0.1 mSv [90% confidence interval: −0.2 to 0.3 mSv]) (Fig. 2). Also, the two groups had equivalent values of CNR (13.5 [95% confidence intervals: 11.8–15.1] vs. 12.8 [95% confidence intervals: 11.3–14.2] and SNR (15.5 [95% confidence intervals: 13.5–17.4] vs. 13.8 [95% confidence intervals: 12.1–15.4]). Also, equivalence was found in tube current, tube voltage and dose-length product (Table 3).

**Table 3:** CT technical characteristics and radiation dose estimates in the study patients.

	Cardiac and lung CT (Gr.A; n = 55)		Cardiac CT only (Gr. B; n = 55)		Mean difference	90% CI
	Mean	95% CI	Mean	95% CI		
Effective radiation dose (mSv)	1.5	1.2–1.7	1.4	1.1–1–6	0.1	−0.2 to 0.3
Contrast-to-noise ratio	13.5	11.8–15.1	12.8	11.3–14.2	0.7	0.1 to 1.5
Signal-to-noise ratio	15.5	13.5–17.4	13.8	12.1–15.4	1.7	0.4 to 2.9
Tube current (mAs)	330	310–349	305	288–321	25	11 to 45
Tube voltage (kVp)	111	109–112	106	106–109	5	−1 to 12
Dose-length product (mGy · cm)	107	90–123	100	81–118	7	−2 to 15

CI = confidence interval; CT = computed tomography.

### Inter-observer agreement

**Figure 3:**
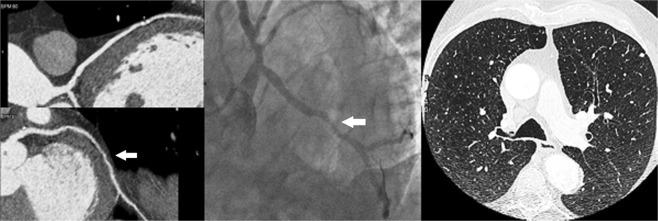
A case of coronary artery disease detection. Cardiac CT revealed a significant 80% stenosis (*white arrow*) of the left circumflex artery (left panel), which was confirmed (*white arrow*) at invasive coronary angiography (middle panel). CT images of the lungs did not show any pulmonary nodule (right panel).

Inter-observer agreement for image quality grading was high for both groups (0.85 for Gr.A and 0.81 for Gr.B). Overall, 25 significant coronary stenoses (>70% reduction of vessel diameter) were found with CT scanning in 20 subjects (9/55 cases of Gr.A vs 11/55 cases of Gr.B, NS). Inter-observer agreement for coronary stenosis >70% was excellent in the two groups (0.89 for Gr.A and 0.90 for Gr.B). By protocol, the 20 patients who had evidence of >1 significant stenosis underwent ICA which confirmed 23/25 stenoses (92%) (Fig. 3**)**. Two critical stenoses detected in a Gr.B patient at time of cardiac CT were found to be non significant at ICA. Agreement between cardiac CT and ICA for detection of coronary stenoses >70% was excellent (0.95 for Gr.A and 0.93 for Gr.B).

### Pulmonary CT evaluation

**Figure 4:**
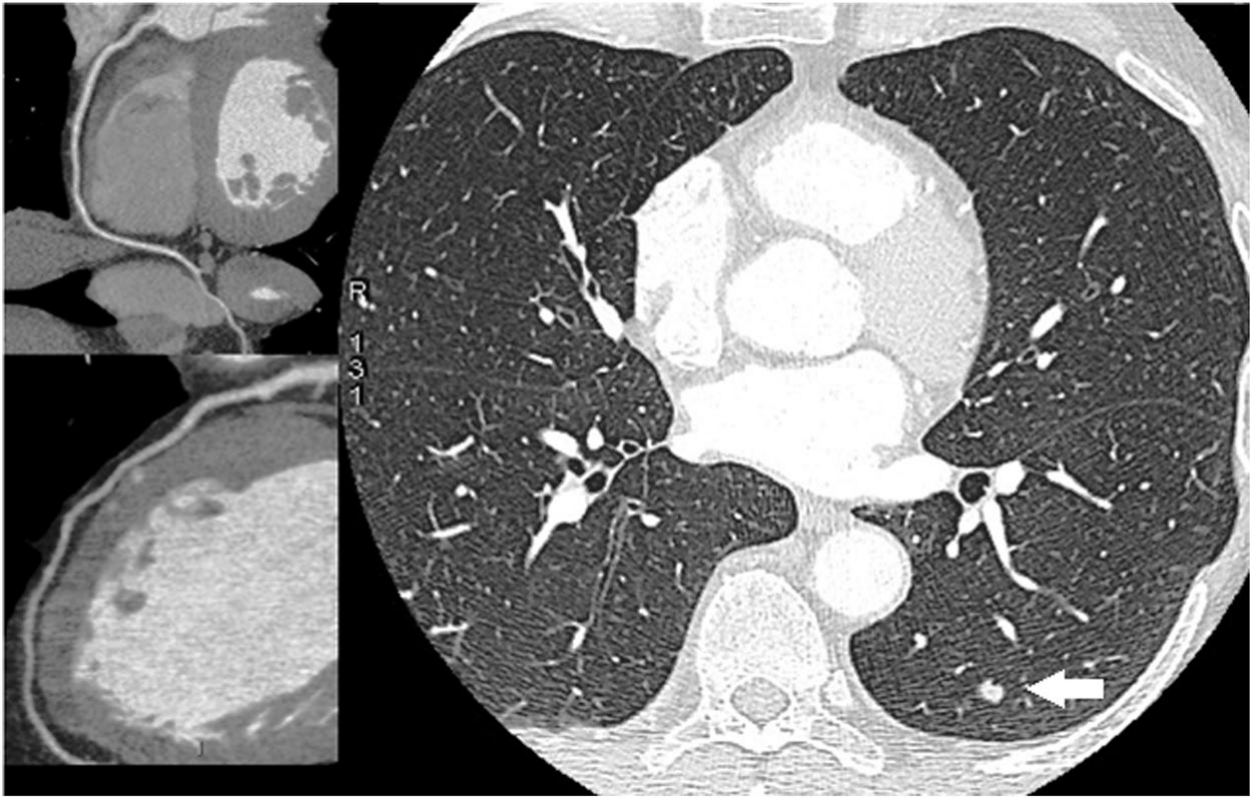
A case of coronary artery disease and lung cancer screening. Ultra-low-dose CT images of the lungs showed a 2 mm pulmonary nodule (*white arrow*) in the left upper lobe (right panel). The cardiac CT revealed normal right coronary artery (left upper panel) and left coronary artery (left lower panel).

**Figure 5:**
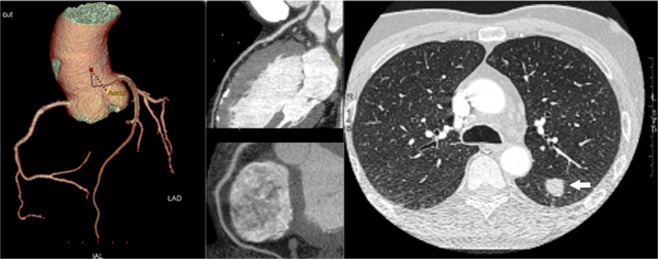
A case of coronary artery disease and lung cancer detection. CT images of the lungs showed a 20 mm pulmonary nodule (*white arrow*) in the left lobe (right panel). The patient underwent a complete diagnostic work-up, including positron emission tomography, and eventually underwent surgical resection. The cardiac CT revealed normal left coronary artery and right coronary artery (left panels).

Pulmonary nodules were detected in 7 of the 55 Gr. A subjects who had simultaneous lung and cardiac CT evaluation (Table 4). Three subjects had evidence of solid nodules <6 mm. These nodules were deemed as ‘negative’ on the basis of the NCCN recommendations (Fig. 4). Four cases had solid nodules >6 mm (range: 6–11 mm). In 2 of them, follow-up thorax CT was scheduled. Two patients, conversely, had a complete diagnostic work-up, including positron emission tomography, and eventually underwent surgery (Fig. 5). Lung cancer (adenocarcinoma) was confirmed by histological study after tumor resection in both cases. No pulmonary nodules could be detected in the 55 Gr.B who underwent cardiac CT only.

**Table 4:** Clinical, diagnostic and therapeutic features of patients with evidence at CT scanning of pulmonary nodules.

N.	Age	Sex	Smoking	Indication to Cardiac CT	Cardiac CT	Lung CT	Therapy
1	67	F	Current	Multiple risk factors	No stenosis	Single nodule 4 cm	Follow-up CT scan at 12 month
2	59	F	Current	Positive exercise stress test	LCx: multiple plaques but no stenosis	Single nodule 6 mm	Follow-up CT scan at 3 month
3	69	M	Current	Multiple risk factors	No stenosis	Single nodule 2 cm	PET-CT and surgery
4	61	M	Former	Family history of CAD	No stenosis	Single nodule 5 mm	Follow-up CT scan at 3 month
5	77	M	Current	LV systolic dysfunction (EF: 45%)	Diffuse atherosclerosis but no stenosis	Single nodule 7 mm	Follow-up CT scan at 3 month
6	69	M	Current	Multiple risk factors	Diffuse atherosclerosis but no stenosis	Single nodule 14 mm	PET-CT and surgery
7	44	M	Current	Family history of CAD	LAD: atherosclerosis but no stenosis	Single nodule 5 mm	Follow-up CT scan at 3 month

CAD = Coronary artery disease; CT = computed tomography; LCx: Left circumflex artery; EF = Ejection fraction; F = Female; M = Male; LAD = Left anterior descending artery; LV = Left ventricle; PET = Positron emission tomography.

## Discussion

Cardiac CT offers an accurate anatomical evaluation of CAD comparable to ICA^5^. For this reason, coronary CT angiography has rapidly become an alternative to ICA for CAD assessment and the new generation scanners are now considered to have high diagnostic performance for detection of significant coronary stenosis in different populations^2^. The low cost and high sensitivity of cardiac CT makes it the non-invasive test of choice in the evaluation of stable angina^2^. This has now been ratified in national guidelines with National Institute for Health and Care Excellences (NICE) recommending cardiac CT as the first-line investigation for all patients presenting with angina pectoris in whom CAD is therefore suspected^1^. Also, randomised controlled trials have demonstrated that cardiac CT improves diagnostic certainty when incorporated into chest pain pathways, particularly in those with high cardiovascular risk scores^16^.

Lung cancer is one of the most common forms of malignancy in both men and women^17^. Early diagnosis is crucial in an attempt to decrease mortality. To this end, large studies have shown that CT screening in high-risk individuals (i.e. current or former heavy-smokers aged 55 to 74 years) yields a 20% decrease in mortality for lung cancer^18^. On the basis of these evidences, in recent years, several expert North American bodies have issued guidelines for screening high-risk populations, that at present have not been adopted worldwide^19^. Specifically, the U.S. Preventive Services Task Force has awarded a Grade B draft recommendation for annual screening with CT in current and former heavy smokers with an age ranging between 55 and 80 years^4^.

Given that thoracic and cardiac disease etiological factors and disease processes overlap^20^, it is not surprising that heart and lung diseases might coexist in the same middle age or older subjects, particularly in current or former smokers with atherosclerosis who are more likely to develop lung cancer^21^. In these patients, the incidental detection of lung comorbidities at time of cardiac CT is common. A systematic review identified a prevalence of 16% for clinically significant lung findings^22^. Similarly, in the SCOT-HEART study, the rates of noncardiac findings was 10%^23^. Most recently, in a series of 2,479 CTs, Robertson *et al*. have found lung nodules in 358 patients (13.9%)^24^. One should consider, however, that lung cancer screening can not be reliably performed by means of conventional cardiac CT, whose reconstructing images include a limited field-of-view of lung parenchyma in order to improve spatial resolution of the coronary arteries^25^. As a consequence, high risk patients requiring CAD and lung cancer screening usually undergo two distinct CT examinations in two different settings. Accordingly, the results of our study are of major importance as they show that a new protocol of CT scanning allows simultaneous CAD and lung cancer screening during one CT session only. Noteworthy, our findings demonstrate that cardiac and lung CT examinations can now be performed concomitantly without affecting negatively the assessment of coronary artery segments or exposing patients to higher radiation doses as compared with standard cardiac CT scanning.

This study was planned and performed as a pilot trial in preparation for a larger multicenter trial. As a consequence, a number of limitations should be acknowledged.

A limitation lies on the relatively small sample size. Also, a cross-over study design would have been more helpful than an open label randomised study design in order to compare different CT protocols in the same patient. The novel CT protocol was not compared with standard lung CT acquisitions. Pulmonary evaluation was done only in a subset of patients and, therefore, there was no valid comparison between the two groups. Thus, the results of our study do not allow one to draw any definitive conclusion about the diagnostic accuracy of the novel combined lung and cardiac CT protocol with respect to conventional lung scanning. Patients did not undergo either CT evaluation or ICA and, therefore, correlation between the two examinations could not be assessed. Previous work, however, has already ascertained that these two diagnostic techniques yield a similar diagnostic accuracy for CAD^26^. Moreover, fast CT protocols have a lower diagnostic ability to detect pulmonary nodules in obese subjects due to the fact that a greater body mass index is associated with a greater image noise^27^. Our patients contained no subsolid or semisolid lesions, thus findings remain limited to solid lesions. Also, our study does not address findings other than pulmonary nodules >2 mm. As a consequence, no conclusions can be drawn on the use of simultaneous cardiac and lung CT scanning for the evaluation of interstitial lung disease or other pulmonary or cardiac conditions in the study population. A further limitation is constituted by the lack of a control group. As a consequence, our preliminary findings might not apply in subset of patients with clinical characteristics different from those of our study population (i.e. current or former heavy smokers whose age range between 55 and 79 years).

In conclusion, this pilot randomised study suggests that simultaneous lung cancer screening and coronary artery evaluation by means of a new CT protocol is feasible and safe. This approach has the potential to increase the cost-effectiveness ratio of coronary CT in subjects who are current or former heavy smokers. Indeed, the new protocol allows a comprehensive – rather than partial - evaluation of both cardiac and lung regions during one CT scanning only, thus avoiding to double the doses of radiation and contrast dye which are used when two distinct examinations are performed to study the coronary arteries and the pulmonary areas. Future randomized multicenter trials should confirm these preliminary results and provide more in-depth information on the best CT protocol for simultaneous CAD and lung cancer screening.

## Data Availability

The datasets generated and/or analysed during the current study are available from the corresponding author on reasonable request.
